# Novel potential therapeutic targets of alopecia areata

**DOI:** 10.3389/fimmu.2023.1148359

**Published:** 2023-04-19

**Authors:** Wen Xu, Sheng Wan, Bo Xie, Xiuzu Song

**Affiliations:** ^1^ School of Medicine, Zhejiang University, Hangzhou, China; ^2^ Department of Dermatology, Hangzhou Third People’s Hospital, Affiliated Hangzhou Dermatology Hospital, Zhejiang University School of Medicine, Hangzhou Third Hospital Affiliated to Zhejiang Chinese Medical University, Hangzhou, China

**Keywords:** alopecia areata, immune cells, interleukins, gut microbiota, therapeutic targets

## Abstract

Alopecia areata (AA) is a non-scarring hair loss disorder caused by autoimmunity. The immune collapse of the hair follicle, where interferon-gamma (IFN-γ) and CD8+ T cells accumulate, is a key factor in AA. However, the exact functional mechanism remains unclear. Therefore, AA treatment has poor efficacy maintenance and high relapse rate after drug withdrawal. Recent studies show that immune-related cells and molecules affect AA. These cells communicate through autocrine and paracrine signals. Various cytokines, chemokines and growth factors mediate this crosstalk. In addition, adipose-derived stem cells (ADSCs), gut microbiota, hair follicle melanocytes, non-coding RNAs and specific regulatory factors have crucial roles in intercellular communication without a clear cause, suggesting potential new targets for AA therapy. This review discusses the latest research on the possible pathogenesis and therapeutic targets of AA.

## 1 Introduction

**Figure 1:**
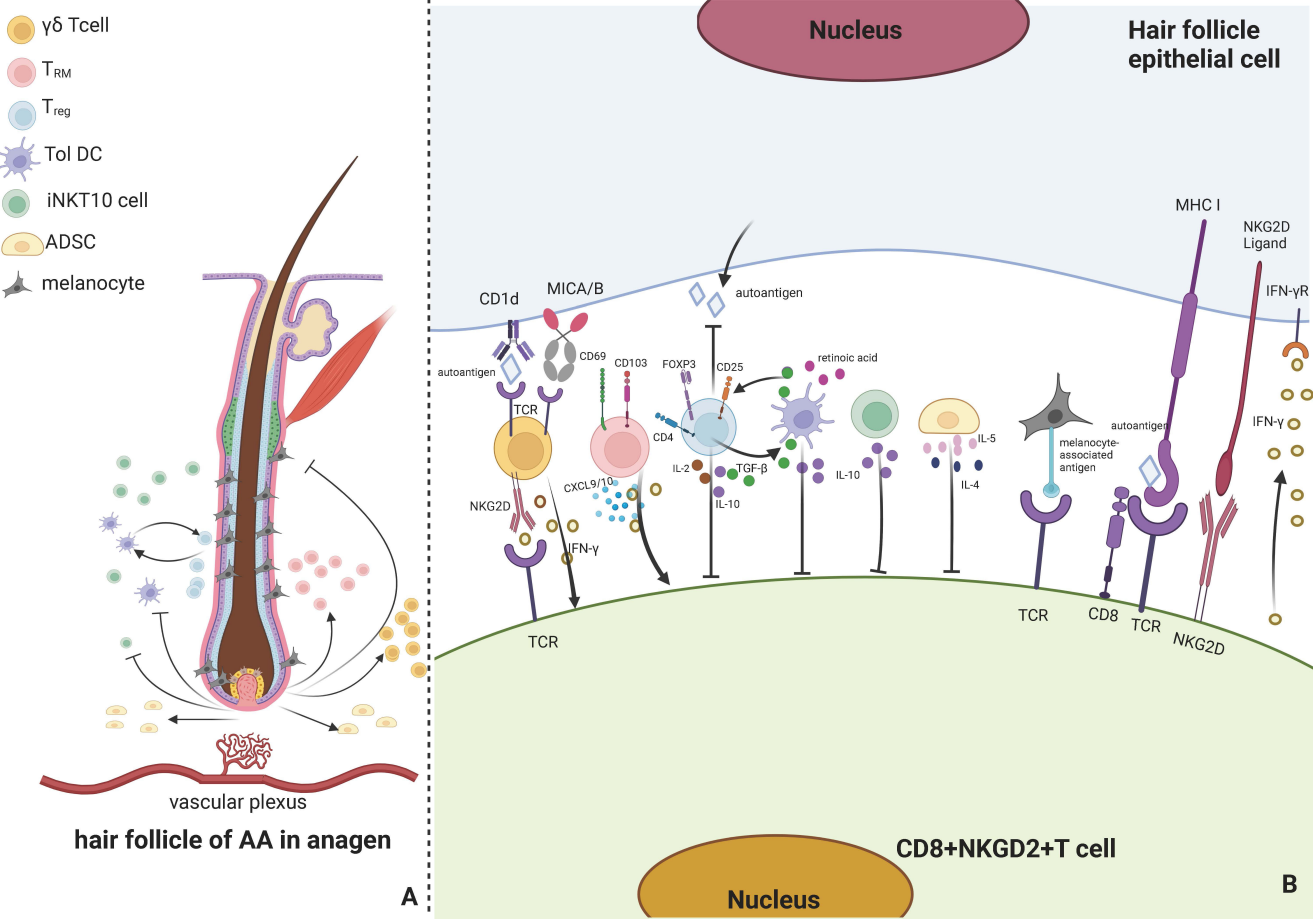
Immune responses involved by associated cells around AA hair follicles. **(A)** presents that the cells involved in the immune response of AA hair follicles in anagen primarily include γδ T cells, TRM cells, T_reg_ cells, Tol DCs, iNKT 10 cells, ADSCs, and melanocytes. When immune privilege collapses and self-antigens are exposed, AA is produced and has various effects, including activating iNKT10 cells, γδ T cells, and T_RM_ cells in the vascular plexus around the hair follicle and suppressing Tol DCs, T_reg_ cells, ADSCs, and melanocytes. Tol DC and T_reg_ cells can mutually activate. **(B)** indicates that TCR on γδ T cells can bind to CD1d and MICA/B in hair follicle epithelial cells, and NKG2D on its surface can bind to CD8^+^ T cells, release IFN-γ, and activate CD8^+^ T cells. T_RM_ can release CXCL9 and IFN-γ and can express CD69 and CD103. T_reg_ cells can express FOXP3, CD25, and CD4, produce IL-2, IL-10, and TGF-β, suppress CD8^+^ T cells, inhibit the autoantigen production on hair follicle epithelial cells, and activate Tol DCs. Tol DC can release retinoic acid, TGF-β, and IL-10, promote T_reg_ cells and inhibit CD8^+^ T cells. iNKT 10 cells can release IL-10 to inhibit CD8^+^ T cells, and ADSCs can release IL-4 and IL-5 to inhibit CD8^+^ T cells. Hair follicle melanocytes can bind to CD8^+^ T cells via melanin-associated antigens and be attacked by T cells. Hair follicle epithelial cells can bind to CD8^+^ T cells and activate CD8^+^ T cells to release INF-γ. Created with Biorender.com.

Alopecia areata (AA) is a non-scarring hair loss disorder that affects about 2% of the population, regardless of race, gender, or age (1, 2). The symptoms and signs of AA vary depending on the severity of the condition, from patchy hair loss to diffuse hair involvement on the scalp or the whole body (3, 4). Most AA patients experience unpredictable relapses and remissions (5). Studies show that it lowers the quality of life of patients and may lead to emotional disorders such as depression and anxiety (6–8). Further associations between AA and certain inflammatory and metabolic diseases have been observed, increasing the probability of developing them (9, 10); however, causality remains to be established and the exact pathogenesis of AA is still to be discovered. The pathogenesis of AA is driven by immune factors, as the current mainstream view suggests (11). Genetic factors may also contribute. Variants in PRDX5 and STX17, genes expressed in the hair follicle, can impair its immune privilege and induce autoimmune responses leading to hair loss (12). A recent study linked mutations in KRT82 to the loss of hair follicle immune privilege and CD8+ T cell infiltration (13). These mutations may contribute to the loss of immune privilege of the hair follicle, which may be a contributing factor of AA development (14, 15). Current conventional treatments and immunobiological therapies do not provide the durable effect or permanent reversal of AA (16, 17). Therefore, it is essential to understand AA pathogenesis comprehensively and identify new therapeutic targets. CD8^+^ T cells have been considered the culprit in AA (18), but with the development of cellular and molecular biology, it was discovered that they are not the only driver of AA. In addition, studies have revealed that numerous unconventional, such as gamma delta (γδ) T cells, interact with CD8+ T cells and influence AA pathogenesis (19, 20), as shown in **Figure 1A**. Simultaneously, ADSCs, gut microbiota, hair follicle melanocytes, and non-coding RNA may contribute to the development of AA. However, the causal relationship between these factors and AA is unclear. This review will provide an overview of the relevant studies.

## 2 γδ T cells

According to different receptor types, T cells can be classified into two subtypes: αβ T and γδ T cells (21). γδ T cells constitute fewer than 5% of the population and are uncommon in the T cell compartment of the blood and secondary lymphoid organs. Still, they are abundant in the skin epithelial tissues (22). Antigen-presenting cells (APCs) detect antigens with the help of the major histocompatibility complex (MHC) molecules on their surfaces. Unlike most human T cells that are MHC-restricted, γδ T cells do not need MHC activation to recognize antigens, as they are major players in innate immune responses along with inflammasomes and INF γ (23–25). Additionally, γδ T cells produce cytokines in the periphery and can be recruited to the lesion where they accumulate (26). This process occurs more rapidly than αβ T cell activation. The ability of γδ T cells to get activated without specific T cell receptor (TCR) ligands renders them potent early inflammasome inducers (27, 28). Depending on their cytokine production, γδ T could be classified into three subsets: IL-17 producers, IFN-γ producers, and IL-4 producers (29). High levels of IFN-γ can trigger skin-related autoimmune disorders via its expression. A recent study revealed that γδ T cells could also express natural killer group 2D (NKG2D) (30). AA patients’ hair follicles highly express CD1d and human major histocompatibility complex class I chain-related gene A (MICA), which can bind to MHC-I and NKG2D receptors and promote AA occurrence (31, 32), as shown in **Figure 1B**.

The researchers compared the number and activation state of T cells in the lesional and non-lesional scalps of AA patients to the scalps of healthy controls. Uchida et al. (33) indicated that the number of γδ T cells was significantly higher in the scalp of AA patients than in healthy controls. γδ T cells promote inflammation, resulting in high NKG2D and IFN-γ expressions. They discovered that γδ T cells increased in both AA non-lesional and lesional scalps, primarily in and around the hair follicle, presenting that γδ T cells play a role in the early development of AA and are agonists for the onset of AA, thereby facilitating the identification of future treatment targets. Additionally, their research discovered (34) that human γδ T cells have a physiological stress sentinel role, wherein their hyperactivity can promote autoimmunity against stressed HF overexpressing CD1d and MICA, which is essential for the early onset of AA. Therefore, inhibiting γδ T cells in the hair follicle could be a proper neoadjuvant method for treating acute AA.

Research on γδT cells has shown promise in the treatment of various diseases. Targeting immune checkpoints around tumors can improve the function of γδT cells within the tumor microenvironment, leading to enhanced proliferation, activation and cytotoxicity (35). Recent studies have also identified CFTR, a regulator of chloride and ion channels primarily studied in epithelial cells and expressed on γδ T cells, as a negative regulator of IFN-γ production and antitumor immunity (36). Both genetic overexpression and pharmacological activation of CFTR can reduce IFN-γ release by peripheral γδ T cells. Thus, targeting γδ T cells around hair follicles may hold significant potential for treating of AA.

## 3 Tissue-resident memory T cells (T_RM_ cells)

T_RM_ cells are long-lived lymphocytes that remain in tissues, particularly in the skin, making it an ideal location for rapidly responding to infection (37). They are essential immunological components that provide the organism with an immediate and precise response to pathogen reinfection or antigen re-exposure of peripheral tissues and are involved in early inflammation (38, 39). T_RM_ cells can continuously monitor and inspect tissues. When suspected antigens and pathogens are discovered, T_RM_ cells are activated (40, 41). It can mediate the immunological responses of other immune cells and tissue (42). Additionally, it may produce cytokines, such as IFN-γ (43), and release cytotoxic substances, such as granzyme B and perforin (44). T_RM_ cells are crucial in tissue differentiation and the early period of an inflammatory immune response. Tissue-specific signals influence its differentiation (45). T_RM_ cells are predominantly generated by T cell-mediated immunological responses (46). Simultaneously, T_RM_ cells can recirculate memory T cells through the blood and lymphoid organs (47). However, the number of T_RM_ cells decreases during recirculation. Recent research has demonstrated that T_RM_ cells can also be created during autoimmunity. T_RM_-mediated autoimmunity is extremely devastating because autoantigens cannot be eliminated and continue to stimulate self-reactive T_RM_ cells (48).

NKG2D^+^ and CD8^+^ T cells are responsible for the advancement of AA, inducing a collapse of immunological response by activating T cells and dendritic cells and causing perifollicular T cells to produce IFN-γ, CXCL9, and CXCL10 (5), as shown in **Figure 1B**. Duca et al. (49) discovered that an AA scalp had more CD103^+^CD69^+^ T_RM_ cells than a non-lesional scalp via TCR sequencing of CD8 T cells. Notably, Janus kinase inhibitors (JAKi)-treated AA patients are susceptible to relapse during the off-drug interval. A study revealed a rise in CD103^+^CD69^+^ T_RM_ cells in patients who relapsed (50). This indicates that T_RM_ cells may be associated with AA and might be a significant aspect of understanding why relapse arises in JAKi-based therapy for AA, making it a good target for more research.

Recent studies have highlighted the potential role of reducing perifollicular T_RM_ cells in the treatment of AA. Fatty acid-binding proteins 4 and 5 (FABP4 and FABP5) have been shown to play critical roles in maintaining the lifespan and function of T_RM_ cells. Deficiency in these proteins impairs the uptake of exogenous free fatty acids by T_RM_ cells and reduces their long-term survival *in vivo*, without affecting central memory T cells in lymph nodes (51). Additionally, research has demonstrated that tissue-resident memory T helper 17 (T_RM_17) cell proliferation and retention in skin requires interleukin-23 (IL-23), and blocking IL-23 can inhibit T_RM_ cell proliferation. This method has already been applied to the treatment of psoriasis and holds potential for future use in AA (52).

## 4 Regulatory T cells (T_reg_ cells)

T_reg_ cells are immunosuppressive cells that inhibit the immune response of other cells and serve as the principal regulators of self-tolerance (53). Its absence or aberrant function results in the onset of autoimmune disorders (54). T_reg_ cells are a subset of T cells with a cellular profile that express FOXP3, CD25, and CD4 (55). Dendritic cells, specifically tolerogenic dendritic cells, may limit adaptive immunity activation by promoting T_reg_ cell differentiation into suppressive subtypes (56). According to a study, transferring T_reg_ cells from skin-draining lymph nodes of mice with normal hair to AA-affected ones reduced the development of generalized AA and site-specific alopecia in the latter but did not promote hair regrowth (57). The study discovered that the efficacy of IL-2 in treating of AA in mice is limited. However, several studies have demonstrated that modest doses of IL-2 injected subcutaneously into AA patients recruit CD4^+^CD25^+^FOXP3^+^ T_reg_ and induce effective hair regeneration (58). This may be because the AA process in humans and mice differs. There is evidence that T_reg_ cells can directly influence hair follicular circulation in AA mice models. Hamed et al. discovered that a lack of T_reg_ cells around the hair bulb leads to a reduced transition from telogen to the anagen phase, reduced hair regeneration, and reduced follicular stem cell differentiation, with an increase in the proportion of the telogen phase (59). A study exhibited that CD80CD86 double-negative C57BL/6 mice are prone to decreased CD4^+^FOXP3^+^ T_reg_ cells, causing spontaneous AA (60). Additionally, studies have confirmed a decrease in FOXP3^+^CD39^+^ T_reg_ cells in the peripheral blood of AA patients (61). However, studies have revealed that TGF-β secreted by T_reg_ cells is higher in scalp and blood samples of AA patients than in healthy controls, and IL-17 is also elevated, as shown in **Figure 1B**. The researchers speculate that T_reg_ cells are more inclined to differentiate into IL-17a-producing cells (62). Phase 2a randomized clinical research revealed dupilumab’s efficacy in treating of AA patients (63–65). It has been claimed that dupilumab stimulates hair development by enhancing T_reg_ function, which is decreased in typical Th2 inflammation (66, 67). Although the mechanism by which T_reg_ cells inhibit AA remains unclear, in the AA mouse model, stimulation of myeloid-derived suppressor cells exosomes (MDSC-Exo) to increase T_reg_ cells *in vivo* can reduce lymphocyte proliferation and activity around the hair follicle and promote hair growth (68), indicating that T_reg_ cells are effective therapeutic targets.

## 5 Tolerogenic dendritic cells (Tol DCs)

Dendritic cells are well-known immunologically presenting cells, and immature dendritic cells predominantly produce immunomodulatory factors to mediate central and peripheral immune responses (69). Tol DCs are a subtype of dendritic cells that promote T_reg_ cell activity, reduce Th1 and Th2 inflammation, and inhibit effector T cells (70). Tol DCs may acquire tolerance to various immune cells implicated in autoimmunity through contact-dependent interactions and pleiotropic cytokines and metabolite production that may treat autoimmune illnesses (71, 72). Therapies based on the injection of Tol DCs exhibited promising results as alternatives to immunomodulators in some chronic inflammatory diseases and organ transplantation (73, 74) because Tol DCs can reduce autoimmune responses without spreading widespread immunosuppression. Tol DCs may be generated for therapeutic applications by adopting dendritic cell vaccination protocols. According to a survey (75), dendritic cells were cultured with drug resistance-inducing medicines (rapamycin or dexamethasone) and cytokines (transforming growth factor-beta (TGF-β) and interleukin-10 (IL-10)) to develop a drug-resistant phenotype *in vitro*, as shown in **Figure 1B**. It is efficient to reintroduce these “induced” Tol DCs into the body. Epigenetic indicators, like DNA methylation and histone modifications, after translation, may also be used to influence cell phenotype and function, either directly or through various studies highlighting the significance of numerous epigenetic mechanisms in the establishment of DC tolerance. Tol DC is generated during *in vitro* differentiation of human monocytes (76, 77). Prostaglandin E2 (PGE2) has altered the ability of Tol DC to suppress CD8^+^ T cell proliferation *in vitro* by inducing DNA Methyltransferase 3A (DNMT3A) upregulation, thereby mediating immunogenic gene methylation and silencing (78). Depending on the induction method, these cells exhibit an intermediate phenotype with traits derived from immature and activated adult dendritic cells, including alterations in migratory activity, anti-inflammatory cytokine production, and the kind of T cell-induced tolerance response (79, 80). These DCs must be loaded with disease-specific autoantigens to develop therapeutic DCs that target specific autoimmune disorders (81). It is now being utilized in phase I and II clinical studies for illnesses, such as type I diabetes and multiple sclerosis, where it has demonstrated encouraging outcomes (82).

Given the abundance of Tol DCs within the skin, epigenetic modifications and therapies targeting epigenetic mechanisms may offer novel avenues for modulating Tol DC adaptability in AA. Additionally, further investigation into hair follicle-specific antigens present in AA, such as keratins and melanocyte antigens, may prove fruitful. However, it should be noted that no studies to date have reported on the use of Tol DCs loaded with autoantigens for the treatment of AA.

## 6 Invariant natural killer T (iNKT) cells

The association between AA and CD8^+^NKG2D^+^ effector T cell drive has been well-documented. However, recent studies have revealed that iNKT cells express NKG2D receptors and rapidly release cytokines in response to early stimulation, possibly making them necessary for Th cell polarization by creating a cytokine environment for conventional Th cells (83). iNKT cells are CD1d-restricted, lipid-reactive T cells with predominantly immunomodulatory properties to produce cytokines and chemokines and modulate other immune cells (84). Additionally, iNKT cells can provide cytotoxic immune responses via cytotoxic granules and death receptor pathways (85). In arthritis, some iNKT are promoted while others are suppressed. These findings suggest that iNKT-mediated regulation in autoimmune diseases is dependent, at least in part, on their ability to regulate macrophage inflammatory profiles (86).

According to Th cell nomenclature, iNKT cells are categorized as iNKT1, iNKT2, iNKT10, and iNKT17 based on cytokine (87). In chronic inflammation, iNKT10 cells, primarily in adipose tissue, express the transcription factor E4BP4 and release IL-10, as shown in **Figure 1B**. Chen et al. activated adipose tissue iNKT10 cells by injecting GalCer, a traditional activator of iNKT cells, subcutaneously into animals fed a high-fat diet, boosting M2 macrophage polarisation and alleviating chronic inflammation in obese adipose tissue (88). Ghraieb et al. demonstrated that the agonist-GalCer could block AA in iNKT10 cells using a humanized mouse model of AA, allowing their expansion to produce IL-10 and activate memory iNKT cells, thereby demonstrating the protective effect of iNKT cells against AA *in vivo* (89). Targeting iNKT cells as a treatment for autoimmune diseases such as type 1 diabetes has been shown to have the potential to help determine the state or severity of an immunological disease (90, 91).

## 7 ADSCs

ADSCs, mesenchymal stem cells (MSCs) in the stromal vascular fraction (SVF) of adipose tissue, are more abundant in adipose tissue, including subcutaneous adipose tissue, than in MSCs of other origins and are an integral part of regenerative medicine (92). These growth factors influence neighboring cell’s activity and play a crucial role in neovascularization (93). Therefore, injecting autologous fat into the scalp before hair transplantation can enhance blood vessel distribution in the scalp and stimulate hair development (94). In addition to their potential applications in tissue repair and regeneration, ADSCs have substantial immunomodulatory properties, such as regulating inflammatory or autoimmune diseases, such as arthritis, colitis, and other autoimmune disorders (95–97). Several studies have demonstrated that their internal extracellular vesicles (EVs) play an essential role. Jafarinia et al. targeted human ADSCs, ADSCs-EVs, and placebo via tail vein injection in a mouse model of induced experimental autoimmune encephalomyelitis. They demonstrated that intravenous ADSC-EV enhanced the anti-inflammatory capacity of T cells by decreasing their proliferative capacity and leukocyte infiltration (98). A recent study revealed that in an *in vitro* model of neuropathy, ADSCs boost anti-inflammatory effects and IL-4 and IL-5 expressions, thereby dampening the immune system (99), as shown in **Figure 1B**. ADSCs have regulated intracellular signaling pathways in adjacent cells and protect the organism by secreting substantial quantities of cytokines, growth factors, and antioxidant substances into their milieu (100). However, the exact mechanism remains unknown, and numerous studies suggest that ADSCs are crucial for preventing cell death caused by oxidative stress (101). ADSCs can improve the anti-aging treatment of senescent cells and animal models of early aging by accelerating the autophagy of mitochondria, eliminating intracellular reactive oxygen species (ROS), and improving mitochondrial quality (102).

Additionally, ADSCs treatment can improve hair loss by promoting mitochondrial division. Anderi et al. established the efficacy and safety of the treatment by injecting 4–4.7×10^6^ autologous ADSVCs into the scalps of AA patients. After three and six months of ADSVCs treatment, all patients had hair regrowth, enhanced hair growth, and a decreased tension test, demonstrating the treatment’s efficacy and safety (103). A randomized, double-blind, controlled clinical trial using adipose‐derived stem cell constituent extract (ADSC-CE) topical solution has the potential to serve as an alternative therapy method for hair regrowth in AGA patients, enhancing hair density and thickness while preserving appropriate treatment safety (104). It cannot be denied that it could prevent AA, although its exact mechanism of action is unknown.

## 8 Hair follicle melanocytes

In clinical practice, most AA patients demonstrate an unexpected phenomenon: white hair in the lesions of balding patients does not fall out. However, almost all black hair has vanished. This is particularly true for early-stage AA sufferers (105). AA patients exhibit a significant reduction in melanocytes (106). Since melanocyte production and melanin synthesis in mature hair follicles occur primarily during the anagen phase, a reduction in melanocytes in the hair follicle may be associated with the early onset of AA.

Bertolini et al. examined lesion sections from balding patients using immunohistochemistry. They discovered that CD8^+^ T cells recognized and attacked the autoantigen presented by follicular melanocytes, providing further evidence that autoantigens are present in follicular melanocytes of balding patients (107). AA has been associated with melanocyte-specific antigens that have yet to be identified. In healthy populations, melanin antigens, such as tyrosinase, MAGE-A3, Melan-A/MART-1, gp100, and NY-ESO-1 may elicit an immune response against CD8^+^ T cells, but they also protect against the formation of melanoma and are persistently overexpressed in humans (108), as shown in **Figure 1B**. In vitiligo, dendritic cells transfer antigenic proteins from normal or stressed melanocytes, such as gp100, which are then recognized by invading T lymphocytes and cause melanocyte death. However, it is unknown what causes melanocytes to perish. The most commonly recognized explanation is that oxidative stress induces an immune response that results in melanocyte death. Melanocyte death may occur via apoptosis, autophagy, autophagic cell death, or ferroptosis when oxidative stress causes high levels of ROS, thereby leading to molecular and organelle dysfunction (109). Kang et al. discovered that oxidative stress could cause apoptosis, which is exacerbated by abnormal calcium accumulation in the mitochondria, and that melanocytes experience mitochondria-dependent apoptosis when oxidative stress increases the expression of transient receptor potential cation channel subfamily M member 2 (TRPM2), allowing a higher calcium influx and consequent cell death (110). Oxidative stress has been connected with melanocyte apoptosis. A meta-analysis indicated that the oxidative marker levels were significantly higher in severe AA patients than in mild to moderate AA patients (111). Multiple studies suggest that oxidative stress may play a substantial role in the pathogenesis of age-associated macular degeneration and melanocyte death (112–114). The AA treatment may include using antioxidant supplements to alleviate oxidative stress or antioxidants in combination with other therapeutic strategies to boost melanocyte oxidative capacity. Several studies (115–117) have demonstrated that antioxidant supplements, such as Yucca, polyunsaturated fatty acids, and carotenoids, preserve melanocytes and improve the therapeutic efficacy of phototherapy in vitiligo patients.

## 9 Gut microbiota

**Figure 2:**
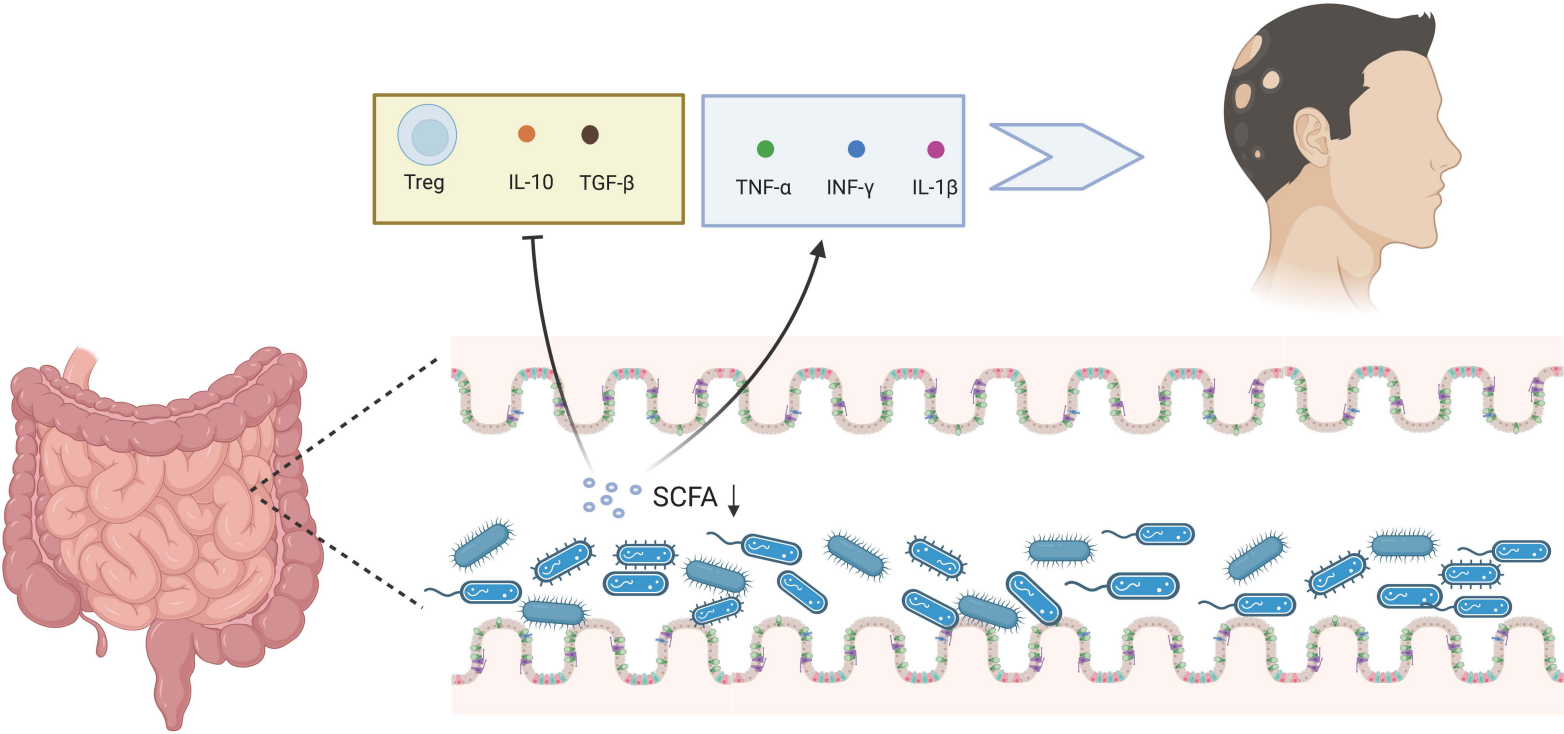
Gut microbiota and AA. The dysregulation of gut microbiota can reduce the SCFA, stimulating the release of TNF-α, IFN-γ, and IL-1β, while suppressing T_reg_ cells and the release of IL-10 and TGF-β, thereby fostering the AA process. Created with Biorender.com.

The gut microbiota is essential for maintaining healthy immune function, regulating T_reg_ cell activity, and monitoring the processing and presentation of antigens (118). Immune-related problems, especially autoimmune diseases, may develop if the gut microbial ecology is imbalanced due to poor food, excessive use of antibiotics, or incompetent breastfeeding (119). Gut microorganisms can regulate specific immune cells and growth factors. Certain probiotics, such as bifidobacteria and segmented filamentous bacteria, can control T-helper 17 cells (120). Bacteroides fragilis surface polysaccharide A binds to toll-like receptor 2 on dendritic cells, inducing T reg cells to generate the anti-inflammatory cytokine IL-10 and promoting immunological tolerance (121). *Clostridium* spp., especially those belonging to clusters IV, XIVa, and XVIII, stimulate CD4^+^FOXP3^+^ T_reg_ cells by producing short-chain fatty acids (SCFAs) (122). Although no microbial medications currently target a specific disease, microbial-based methods for preventing or curing inflammatory autoimmune disorders provide new potential. Most of the metabolites made by microbes in the intestine are SCFAs (123). They are formed when intestinal flora consumes and ferments soluble fibers and oligosaccharides. The intestinal epithelial cells readily absorb them. They are an essential energy source for the bacteria and cells that live in and line the gut. SCFAs control chromatin’s structure in T lymphocytes’ nucleus, making gene products more active (124), as shown in **Figure 2**.

Binding of SCFAs to G protein-coupled receptor 43 (GPR43) has been shown to be essential for the resolution of inflammatory responses (125). While SCFAs exert systemic effects following absorption, local application may be more relevant in the context of AA. Indeed, enemas containing SCFAs have demonstrated efficacy in a subset of patients with distal ulcerative colitis (126). Furthermore, topical application of SCFAs has been shown to promote induction of tissue plasminogen activator in airway epithelial cells via binding to G protein-coupled receptors, reducing fibrin deposition and alleviating chronic sinusitis (127). These findings suggest that topical application of SCFAs to scalp hair follicles may hold promise for the treatment of AA.

## 10 Non-coding RNAs and related regulatory proteins

MicroRNAs (miRNAs) and long non-coding RNAs (lncRNAs) are the most common non-coding RNAs. Various potential miRNAs, lncRNAs, and regulatory proteins have been identified in autoimmune disorders (128). Upregulated miRNAs include miR-210 (129), miR-1246 (130), miR-17 (131), miR-34a (132), miR-101 (133), miR-27b (133), miR-142-3p (134), miR-142-5p (134), and miR-150 (134), while downregulated miRNAs include miR-30b (135), miR-103 (133), miR-2355-3p (133), and miR-186-5p (134) that stimulate their target protein expressions, as shown in **Table 1**. This mismatched miRNA-target interaction causes abnormal T cell activation (134), melanosome autophagy (137), and angiogenesis inhibition (138), thereby damaging hair follicles and contributing to AA. According to the bioinformatics results of Sheng et al., NONHSAT011665-RPS26-has-miR-186-5p may play a role in the control of AA (139). Qi et al. demonstrated that novel treatment for AA might lie in RP11-251G23.5 and RP11-231E19, which may play an important role in the etiology of AA through modulation of the cytokine-cytokine receptor interaction pathway (140). Eomesodermin (Eomes) may play a major role in AA by regulating immune cell infiltration and keratin-forming cell activity, indicating that they are a possible therapeutic target for AA (141). Several JAJ-STAT pathway components downstream of the cytokine-containing chain (known to increase the activity and survival of IFN-producing cytotoxic T cells) were upregulated in human and mouse AA skin. Systemic injection of JAK inhibitors reduced AA development in a mouse model (142, 143). Several open-label phase II clinical studies have demonstrated that oral JAK inhibitor treatment significantly promotes hair regrowth and improves AA with assured safety and dependability (144, 145).

**Table 1:** Functional characterization of the miRNAs in AA patients.

miRNAs	Expression	Target gene	Function	Source	Ref
miR-30b	down	IL2RA/STX17/TNXB	Inhibiting autophagy of melanosomes	Skin lesions in AA patients	(135)
miR-210	up	FOXP3	immunosuppressive functions of T_reg_ cells	Serum of AA patient	(129, 136)
miR-1246	up	TP53	activating immune response.	Serum of AA patient	(130, 136)
miR-17	up	MIR17HG	Promotion of autoimmune disorders	Serum of AA patient	(131)
miR-34a	Up	VEGF	Inhibition of Angiogenesis	Serum of AA patient	(132)
miR-101	Up	–	Enhancing T cell responses	Serum of AA	(133)
miR-27b	Up	–	Enhancing T cell responses	Serum of AA	(133)
miR-103	Down	–	Inhibiting T cell responses	Serum of AA	(133)
miR-2355-3p	Down	–	Inhibiting T cell responses	Serum of AA	(133)
miR-142-3p	Up	AC9	Enhancing T cell responses	Skin lesions in AA patients	(134)
miR-142-5p	Up	SH2D1A	Enhancing T cell responses	Skin lesions in AA patients	(134)
miR-150	Up	IL2RA	Activating immune response.	Skin lesions in AA patients	(134)
miR-186-5p	Down	FOX01	Related to the cell cycle	Serum of AA patient	(134)

Interleukin 2 receptor subunit alpha (IL2RA), syntaxin17 (STX17), tenascin XB (TNXB), forkhead box P3 (FOXP3), tumor protein p53 (TP53), miR-17-92a-1 cluster host gene (MIR17HG), vascular endothelial growth factor (VEGF), adenylate cyclase 9 (AC9), SH2 domain containing 1A (SH2D1A), and forkhead box protein O1-A (FOX01).

## 11 Conclusion and future perspectives

Recent studies have shed light on potential mechanisms for inhibiting the occurrence and development of AA. Activation of immune tolerance cells such as T_reg_ cells and Tol DCs, along with inhibition of immune enhancement cells such as γδ T cells, T_RM_ cells, and iNKT cells may play a role in preventing AA. Additionally, ADSCs and SCFAs from Gut microbiome can improve the inflammatory manifestations of AA, and patients may benefit more with its topical application. Reducing exposure of hair follicle melanocytes’ antigens to CD8+ T cells may also be effective; loading melanocyte-associated antigens through Tol DCs could be a potential approach. Furthermore, noncoding RNAs and regulatory proteins can release small molecules or cytokines such as growth factors or their receptors, opening up new avenues for AA treatment.

## Author contributions

WX, SW, BX, and XS contributed significantly to the analysis and manuscript preparation; WX wrote the manuscript; SW, BX, and XS helped perform the analysis with constructive discussions. All authors contributed to the article and approved the submitted version.

## Glossary

**Table:**

AA	Alopecia areata
IFN-γ	interferon-gamma
ADSCs	adipose-derived stem cells
KRT82	Keratin82
APCs	antigen presentation cells
MHC	major histocompatibility complex
MICA	human major histocompatibility complex class I chain-related gene A
NKG2D	natural killer group 2D
TCR	T cell receptor
MDSC-Exo	myeloid-derived suppressor cells exosomes
T_RM_ cells	Tissue resident memory T cells
T_reg_ cells	Regulatory T cells
TGF-β	transforming growth factor-beta
IL-10	interleukin-10
Tol DCs	Tolerogenic dendritic cells
PGE2	Prostaglandin E2
DNMT3A	DNA Methyltransferase 3A
ROS	reactive oxygen species
JAKi	Janus kinase inhibitors
iNK T	invariant natural killer T
MSCs	mesenchymal stem cells
SVF	stromal vascular fraction
EVs	extracellular vesicles
ADSC-CE	adipose-derived stem cell constituent extract
TRPM2	transient receptor potential cation channel subfamily M member 2
SCFAs	short-chain fatty acids
miRNAs	MicroRNAs
lncRNAs	long non-coding RNAs
IL2RA	interleukin 2 receptor subunit alpha
STX17	syntaxin17
TNXB	tenascin XB
FOXP3	forkhead box P3
TP53	tumor protein p53
MIR17HG	miR-17-92a-1 cluster host gene
VEGF	vascular endothelial growth factor
AC9	adenylate cyclase 9
SH2D1A	SH2 domain containing 1A
FOX01	forkhead box protein O1-A
GPR43	G protein-coupled receptor 43
T_RM_17	T helper 17
